# Clinicopathological features and prognosis of colonic and rectal gastrointestinal stromal tumors: A propensity score matching analysis

**DOI:** 10.3389/fsurg.2022.968585

**Published:** 2022-10-21

**Authors:** Chen Li, Yunwei Lu, Meng-meng Zhang, Hao Wu, Han Li, Ying-Jiang Ye, Kewei Jiang

**Affiliations:** ^1^ Department of Gastroenterological Surgery, Laboratory of Surgical Oncology, Beijing Key Laboratory of Colorectal Cancer Diagnosis and Treatment Research, Peking University People's Hospital, Beijing, China; ^2^ Department of Gastroenterological Surgery, Shandong Provincial Hospital, Cheeloo College of Medicine, Shandong University, Jinan, China; ^3^ Department of General Surgery, The First Affiliated Hospital of Shandong First Medical University, Jinan, China

**Keywords:** gastrointestinal stromal tumors, colonic neoplasm, propensity score matching, stata, rectal neopalsm

## Abstract

**Background:**

Colonic gastrointestinal stromal tumor (cGIST) and rectal gastrointestinal stromal tumor (rGIST) are two rare subtypes of gastrointestinal stromal tumor (GIST). The view that colonic and rectal carcinoma are different is generally accepted; however, whether there is a difference between cGIST and rGIST is still unknown. Here, we aimed to provide evidence for future clinical management and research by comparing the differences between the two types of GIST in the above-mentioned aspects.

**Methods:**

Patients were enrolled from three medical centers in China and published literature was collected following the inclusion and exclusion criteria. Propensity score matching was used to eliminate differences between cohorts.

**Results:**

Between cGIST and rGIST patients, significant differences were observed in age, tumor size, mitotic index, NIH risk category, growth pattern, and symptoms. Adjuvant therapy is used in a high proportion of cGIST patients, and neoadjuvant therapy is used in a high proportion of rGIST patients. Although local resection is the main surgical method in both cohorts, the proportion is higher in cGIST patients. The overall survival of rGIST patients was better than that of the cGIST patients before propensity score matching (PSM). Interestingly, no significant differences in prognosis were observed after PSM.

**Conclusions:**

Although there were significant differences between cGIST and rGIST patients in baseline characteristics, clinicopathological features, treatment choice, and overall survival rate before PSM, no significant differences in long-term survival were observed between the two groups after PSM. In our study, there may be no differences in the tumor entity between cGIST and rGIST.

## Introduction

Gastrointestinal stromal tumor (GIST) is the most frequent subset of gastrointestinal (GI) tract tumors of mesenchymal origin, and the annual incidence was estimated to be approximately 1–1.5 per 100,000 individuals (1). GI tumors can arise anywhere along the GI tract, and frequently occur in the stomach (60%) and the small intestine (30%), but rarely in the rectum (5%), colon (1%–2%), and outside of the GI tract within the abdominal cavity (≤5%) (2–4). The oncological behavior of GIST varies from benign to malignant based on the biological heterogeneity, which includes genetic and site-associated differences (4). Moreover, the location of the tumor is an essential influencing factor in the prognosis of GIST, and a non-gastric tumor location could result in a worse prognosis (5). Approximately 80% of all GISTs bear a mutation of the KIT proto-oncogene (6) and 10% show a PDGFRA mutation (7). GISTs present specific molecular features, and typically stain positive staining for CD117 (95%), CD34 (70%), DOG1 (96%), SMA (25%), desmin (<5%), and S100 (rare) (8, 9). The appearance of Imatinib (tyrosine kinase inhibitor, TKI) has changed not only the strategy of treatment of GIST patients but also the long-term outcomes, especially when Imatinib involves neoadjuvant and adjuvant therapy plus radical resectionm, which has shown promising results in increasing overall survival (10).

Colonic gastrointestinal stromal tumor (cGIST) comprises a rare subset of tumors with an overall frequency of 1%–2% of GIST and approximately makes up 0.1% of all tumors of the large intestine (3, 11). Smaller cGIST are often randomly detected, and larger tumors usually present with lower GI hemorrhage, bowel obstruction, and abdominal pain (12). However, given that cGIST is quite uncommon, published literature that focuses on the topic of cGIST are limited by case reports or small sample retrospective studies.

The rectum is a rare site for GIST, accounting for 3.5%–5% of all GISTs, and 0.6% of all rectal malignant tumors (9). Patients with rectal gastrointestinal stromal tumor (rGIST) usually present with pain, obstruction, gastrointestinal bleeding, and symptoms similar to prostatitis (13). Due to their unique location, rectal GISTs rarely grow to a large size in contrast to stomach and colon GISTs (12.2% vs. 20.6% vs. 20.4%, respectively) (14). Although rGIST is rare and smaller in size, they show a high risk of recurrence and metastasis compared with tumors at other sites (11).

Colorectal cancers ranked third most the commonly diagnosed types of cancer and was the second most common cause of cancer-related death worldwide in the last few years (15). In general, the colon is different from the rectum in embryological origin, anatomy, and function (16–18). Therefore, in an increased number of studies, it was discovered that there are differences in biological hallmarks, clinical behavior, metastatic patterns, and long-term outcomes between primary rectal and colon cancers (19–22). Accordingly, clinical management, such as neoadjuvant and adjuvant therapy are different between colonic and rectal cancers.

To our knowledge, no study has compared the oncological and prognostic differences between cGIST and rGIST. Therefore, in this study, we focused on summarizing the differences and similarities in baseline characteristics, oncological features, clinical management, and follow-up outcomes of cGIST and rGIST through propensity score matching (PSM). Combined with the above-mentioned content and bibliometric analysis, we provide robust evidence of future clinical management and the research landscape of cGIST and rGIST.

## Materials and methods

### Study design and approval

This retrospective cohort study was carried out based on prospectively collected cGIST and rGIST data from a multi-central database, including Peking University People's Hospital (PKUPH), Shandong Province Hospital, and The First Affiliated Hospital of Shandong First Medical University. This study was designed in agreement with the Declaration of Helsinki and approved by the PKUPH Ethics Committee. All relevant procedures were accredited by the Institutional Review Board. The checklist of reporting guidelines in propensity score analysis was implemented (Supplementary Digital Contents) (23).

Regarding literature studies, searches were conducted using the following electronic databases: PubMed, EMBASE, the Cochrane Library, and Web of Science Core Collection (WoSCC), all without publication date restrictions on Jan 1, 2022. The search was performed using the following keywords and terms: (“Gastrointestinal Stromal Tumors” OR “GIST*” OR “Stromal Tumors, Gastrointestinal” OR “Tumor, Gastrointestinal Stromal” OR “Tumors, Gastrointestinal Stromal” OR “Tumors, Gastrointestinal Stromal” OR “Neoplasm, Gastrointestinal Stromal” OR “Neoplasms, Gastrointestinal Stromal” OR “Stromal Neoplasm, Gastrointestinal” OR “Stromal Neoplasms, Gastrointestinal” OR “Gastrointestinal Stromal Tumor” OR “Gastrointestinal Stromal Neoplasm” OR “Gastrointestinal Stromal Sarcoma” combined with “Colon*” OR “Cecum*” OR “Intestine, Large” OR “Appendix” OR “Appendiceal” OR “Rectal” OR “Rectum”). A supplementary literature search was performed through Google Scholar. In this study, we only included publications in English, with no restriction on the data category. All search strategies were peer-reviewed and determined after numerous pre-searches.

### Inclusion and exclusion criteria

The inclusion criteria for cGIST and rGIST patients in the three medical centers were as follows: (1) tumor diagnosed as GIST, which originated from the colon or rectum by postoperative pathological outcomes, and (2) patients who underwent surgery in the three centers mentioned above. Accordingly, patients were excluded if (1) the tumor was not the primary cGIST or rGIST or (2) patients had more than one tumor in the colon and rectum, regardless of whether the neoplasms were GIST or not.

**Figure 1:**
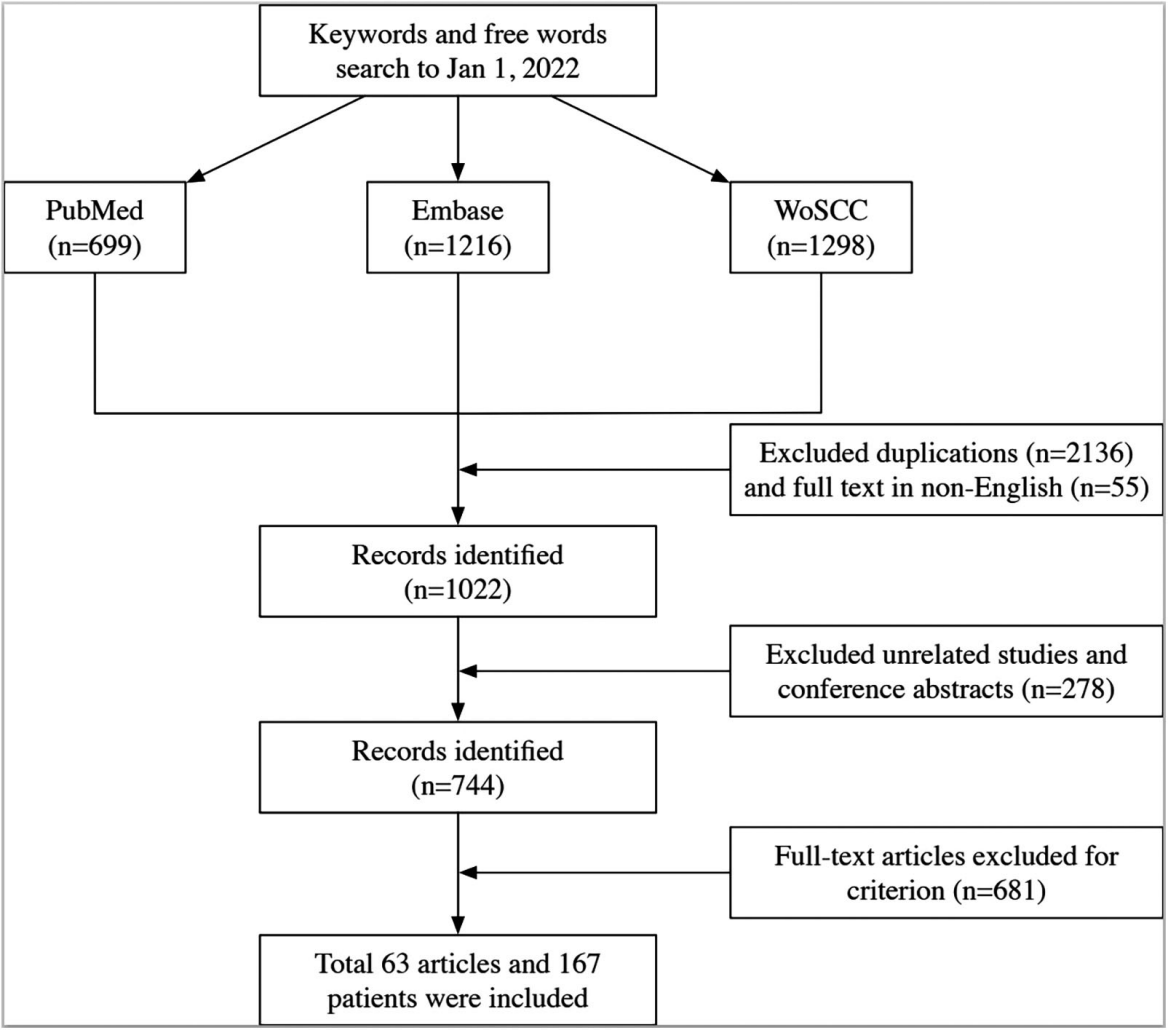
Flow chart of included literature.

Regarding the literature studies, records included articles, reviews, and case reports/series on cGIST or rGIST. Two groups of reviewers (Chen Li/Hao Wu, Yun-Wei Lu/Han Li) independently screened the titles and abstracts after standard selection training, and studies that did not meet the inclusion criteria were excluded. The full text was retrieved when necessary, and disagreements between reviewers were discussed and solved between four reviewers. Moreover, literature-based patient data were registered according to the above-mentioned criteria when the article contained detailed patient data. Furthermore, studies were excluded if they met the following criteria: (1) patients did not undergo surgery; (2) there was a lack of vital information on patients and tumors, such as tumor size. Finally, eligible data were identified. The inclusion and exclusion flow chart is shown in Figure 1.

In this study, overall survival (OS) was identified as the primary outcome and was defined as the time (in months) between initial tumor resection and death. The secondary outcome was progression-free survival (PFS), which was defined as the time (in months) between initial tumor resection and events including recurrence, metastasis, and death. For data without end-point events, the OS and PFS data points were censored at the time of the last follow-up.

### Data collection

The following demographic and clinicopathological characteristics, which were routine variables, were gathered from the multi-central GIST database and literature: age, gender, main complaints, preoperative examinations [including computer tomography (CT), magnetic resonance imaging (MRI), endoscopy and biopsy], neoadjuvant therapy, surgical approach, tumor location, tumor size, growth type, cell morphology, mitotic index (per 50 high power fields), tumor rupture, modified NIH risk category, immunohistochemistry (IHC) results (including CD117, CD34, DOG-1, Ki-67, SMA, S-100, and desmin), postoperative target therapy, and follow-up results. For data obtained from the literature, the title, year of publication, and authors were also extracted.

### Statistical analysis

Continuous variables are expressed as the mean and standard deviation (SD) or median and interquartile range, and categorical variables are presented using frequencies and percentages. Based on the expected values, categorical variables were analyzed using the Chi-square test or Fisher's exact test. The Mann–Whitney U test was implemented to compare continuous variables, which are presented as the median and interquartile range and as the mean ± SD. The Kaplan–Meier method and log-rank test were performed to plot survival curves and to evaluate differences in survival time, respectively. Hazard ratios with a 95% confidence interval were also derived. Statistical significance was defined as *P* < 0.05. Statistical analyses were performed using Stata software (version 16.0; StataCorp LLC).

### Propensity score matching

To minimize the impact of the inhomogeneous distribution of several baseline characteristics and uneven oncological features, patients in this study who were diagnosed with cGIST and rGIST were subjected to PSM, with rigorous adjustment for significant differences in patient clinicopathological characteristics. The “Psmatch2” package in Stata was used to perform a bipartite PSM of the subjects who suffered from cGIST and rGIST. PSM was conducted using a logistic regression model in which the dependent variable was cGIST or rGIST and the independent variables were factors potentially associated with this variable, i.e., age, gender, main complaint, tumor location, tumor size, cell morphology, mitotic index, and modified NIH risk category. Patients were matched based on propensity scores using a caliper width equal to 0.02 of the standard deviation of the logit propensity score. Based on the PSM, patients who were diagnosed with cGIST were matched 1:1 to patients who were diagnosed with rGIST, thereby optimizing the closeness of the matches by assigning the closest matches first.

## Results

Figure 1 shows that after data extraction of 63 studies (17 for cGIST and 46 for rGIST), 53 patients with cGIST and 114 patients with rGIST were included following inclusion and exclusion criteria. From January 2012 to January 2022, 12 cGIST and 51 rGIST patients were identified from Peking University People's Hospital, Shandong Province Hospital, and The First Affiliated Hospital of Shandong First Medical University. To prevent bias of immature surgical skills, all surgeries were performed by senior surgeons. Therefore, a total of 65 cGIST and 165 rGIST patients before matching and 41 cGIST and 41 rGIST patients after matching were included in the final analysis.

The baseline characteristics and tumor features before and after PSM are presented in Table 1. Both before and after matching, there was no significant difference in gender between cGIST and rGIST. The same findings were observed when comparing cell morphology (the most frequently detected cell morphology for the two groups was spindle) and follow-up time between the two groups. Regarding the patients' age, there were 25 patients in the cGIST group and 103 patients in rGIST group who were younger than 60 years of age, while 40 and 62 patients, respectively, were over 60 years of age (*P* < 0.01). Regarding tumor features, a tumor size between 5 cm to 10 cm was the most commonly detected size for both cGIST and rGIST, and there were statistically significant differences between the two groups (*P* < 0.01). The mitotic index of cGIST was mainly less than 5/50 HPF (53.85%, 35/65), and correspondingly, rGIST patients showed the same trend (62.42%, 103/165). Not surprisingly, 69.2% (45/65) of cGIST patients and 54.55% (90/165) of rGIST patients were classified as high risk following the NIH risk category (*P* < 0.01). Furthermore, significant differences were observed in growth type (*P* < 0.01). An extraluminal tumor type was more common in the cGIST group (26/54, 48.15%), while rGIST patients frequently presented with intraluminal tumors (51/94, 54.26%).

**Table 1:** Patient baseline characteristics and tumor features in unmatched and matched cohorts.

	Before Matching		*P*	After Matching		*P*
	cGIST (*N* = 65)	rGIST (*N* = 165)		cGIST (*N* = 41)	rGIST (*N* = 41)	
Gender	*n* = 65	*n* = 165	0.67	*n* = 41	*n* = 41	0.82
Female	30	71		18	17	
Male	35	94		23	24	
Age (yrs)	*n* = 65	*n* = 165	**<0**.**01**	*n* = 41	*n* = 41	1.00
≤60	25	103		16	16	
>60	40	62		25	25	
Size (cm)	*n* = 65	*n* = 165	**<0**.**01**	*n* = 41	*n* = 41	1.00
≤2	14	24		10	10	
>2, ≤5	12	64		9	9	
>5, ≤10	22	70		19	19	
>10	17	7		3	3	
Mitotic Index (per 50 HPF)	*n* = 65	*n* = 165	**0**.**02**	*n* = 41	*n* = 41	1.00
≤5	35	103		29	29	
>5, ≤10	4	25		1	1	
>10	26	37		11	11	
NIH Risk Category	*n* = 65	*n* = 165	**<0**.**01**	*n* = 41	*n* = 41	0.26
Very Low	14	21		10	10	
Low	6	40		5	8	
Intermediate	0	14		0	3	
High	45	90		26	20	
Cell Morphology	*n* = 65	*n* = 118	0.79	*n* = 41	*n* = 23	0.13
Spindle	60	106		40	20	
Non-Spindle	5	12	** **	1	3	
Growth Pattern	*n* = 54	*n* = 94	**<0**.**01**	*n* = 37	*n* = 20	0.82
Intraluminal Type	14	51		10	10	
Extraluminal Type	26	39		16	9	
Others	14	4		11	1	

Clinical symptoms and follow-up results of the tumor before and after matching are presented in Table 2. From the view of clinical manifestation, abdominal pain (7/36 in cGIST patients vs. 22/82 in rGIST patients) and hemorrhage (6/36 of cGIST patients vs. 32/82 of rGIST patients) were major complaints of patients but still existed patients had no complaints of discomfort (11/36 of cGIST patients vs. 7/82 of rGIST patients) (*P* < 0.01). Regarding the surgical approach, most patients (26/32 of cGIST patients vs. 95/158 of rGIST patients) received local resection rather than radical anatomical resection (6/32 of cGIST patients vs. 63/158 of rGIST patients) (*P* < 0.05). In terms of targeted therapy, the proportion of postoperative adjuvant therapy in cGIST patients was significantly lower than that in rGIST (44.44% of cGIST patient vs. 60.13% of rGIST patients), and neoadjuvant therapy showed the same trend (7.41% of cGIST patients vs. 49.37% of rGIST patients).

**Table 2:** Clinical characteristics and follow-up results in unmatched and matched cohorts.

	Before Matching		*P*	After Matching		*P*
	cGIST (*N* = 65)	rGIST (*N* = 165)		cGIST (*N* = 41)	rGIST (*N* = 41)	
Symptom	*n* = 36	*n* = 82	**<0**.**01**	*n* = 23	*n* = 15	1.00
Asymptom	11	7		7	1	
Abdominal Pain	7	22		1	4	
Hemorrhage	6	32	** **	4	4	
Obstruction	2	21	** **	1	6	
Mass	4	0	** **	4	0	
Perforation	6	0	** **	6	0	
Surgery	*n* = 32	*n* = 158	**0**.**02**	*n* = 21	*n* = 39	**0**.**03**
Local Excision	26	95		19	25	
Radical Excision	6	63		2	14	
Target Therapy	*n* = 27	*n* = 79		*n* = 17	*n* = 18	
Adjuvant	12	46	**<0**.**01**	7	9	0.60
Neoadjuvant	2	39	**<0**.**01**	2	8	0.06
Follow-up	*n* = 52	*n* = 148	** **	*n* = 41	*n* = 41	
Time	49.04	46.36	0.71	52.39	41.28	0.23
Outcomes	*n* = 52	*n* = 148	**<0**.**01**	*n* = 41	*n* = 41	0.14
ANED	25	94	** **	21	29	
AWD	3	36	** **	4	5	
DOC	6	5	** **	5	1	
DOD	18	13	** **	11	6	

After propensity score matching, no significant differences were observed among all important variables (age, gender, tumor size, cell morphology, mitotic index). Significant differences were only observed in the surgical approach (*P* < 0.05). Regarding immunohistochemical markers (Supplementary Digital Content S3), CD117 and CD34 showed a higher positive rate in rGIST patients (106/110 and 95/101) compared with cGIST patients (25/32 and 23/29). DOG-1, a specific marker of GIST, was significantly positive in cGIST patients (12/16) and rGIST patients (59/61). In the cGIST patient group, Ki-67 was higher than in the rGIST group (18.5% vs. 9.24%).

**Figure 2:**
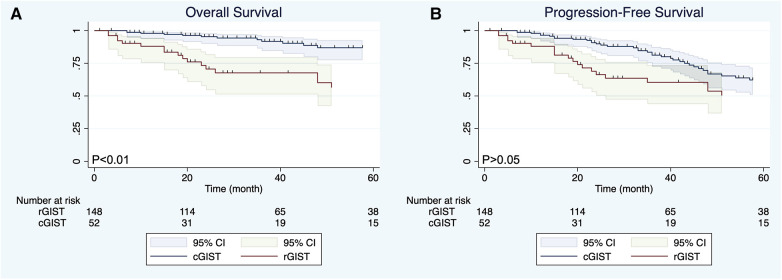
Overall survival (**A**) and progression-free survival (**B**) of cGIST and rGIST before propensity score matching.

**Figure 3:**
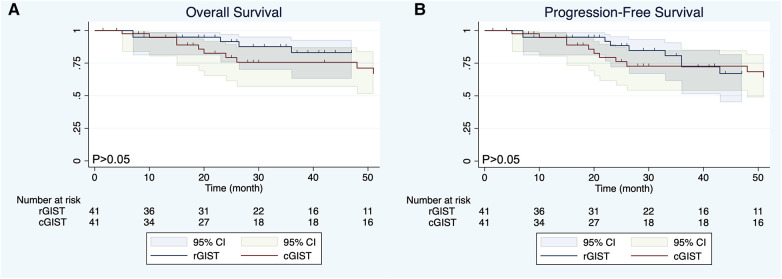
Overall survival (**A**) and progression-free survival (**B**) of cGIST and rGIST after propensity score matching.

Before PSM, the mean follow-up time of the entire matched cohort was 49.04 months (IQR, 3–235 months) and the overall survival (OS) was significant different (*P* < 0.01) between the cGIST and rGIST groups(Figure 2A), although the different was not significant (*P* > 0.05) in progression-free survival (PFS) (Figure 2B). After PSM, 1-year, 3-year, and 5-year, the PFS of cGIST was 94.63%, 65.08%, and 52.88%, and the OS was 94.63%, 75.64%, and 61.46%, respectively. The rGIST data were 94.87%, 72.22%, and 67.06%, and 94.87%, 83.06%, and 83.06%, respectively. When the two cohorts were compared after matching, no statistically significant differences in OS were observed between cGIST and rGIST patients (*P* < 0.001) (Figure 3A) and PFS (*P* < 0.001) (Figure 3B).

## Discussion

The definition of GIST was first proposed by Mazur et al. (24) in 1983. GIST is a group of tumors originating from the gastrointestinal mesenchymal tissue and are characterized by unique histological, immunophenotypic, and molecular genetics. With the development of research, it was found that these tumors are mostly derived from interstitial cells of Cajal (ICCs) or their stem cell precursors, and are associated with activation of mutations in KIT proto-oncogenes (25). GIST can occur anywhere in the digestive tract, but it is mostly found in the stomach and small intestine (26). cGIST and rGIST are rare subtypes of GIST, accounting for only 1%–2% and 3.5%–5% of GIST (27). Due to the limited number of studies on cGIST and rGIST (14, 28–31), the latest GIST guidelines still discuss cGIST and rGIST patients as one group, while ignoring the differences in clinicopathological features and prognosis between the two groups.

In our study, 65 cGIST and 165 rGIST patients from three medical centers in China were enrolled, and existing literature was investigated to compare the differences in baseline and clinicopathological features between cGIST and rGIST patients. The PSM was used to minimize the influence of confounding factors to explore the effect of different primary sites on the prognosis of patients with cGIST and rGIST.

The data showed that patients with cGISTs presented at initial diagnosis with a higher age (a median age of 60) than rGISTs, which was consistent with the findings presented in previous studies (30, 31). Feng et al. (28) initially discussed the relationship between the primary site and the age of GIST. By referring to the results of previous studies (12, 32), they found that the distribution of cGIST positively correlated with the number of ICCs in the colon, and it was speculated that the difference in distribution between different age subgroups might be related to a decline in the number of ICCs and the different rates of decline in colon segments. Recently, in another study (33) from the UK, the same phenomenon was observed that the number of ICCs in the rectum decreased with age. Together, these results provide ideas for exploring the causes of the age difference between cGIST patients and rGIST patients. Gender characteristics of cGIST and rGIST patients have not been determined. Feng et al. (28) reported that cGIST patients were more common in women (57% vs. 43%), and Zhu et al. (14) reported that rGIST patients were more common in men (62.1% vs. 37.9%). On the contrary, Reddy et al. (29) reported that both cGIST and rGIST patients were mostly male patients. Our results are consistent with the study presented by Reddy et al., and we observed that patients in both cGIST and rGIST groups have a male predominance.

In 2001, the National Institute of Health (NIH) developed an evaluation protocol for the clinical behavior of GIST (34), which used tumor size and mitotic index as the main evaluation indicators. However, even the modified NIH risk category (35) divides GIST into gastric and non-gastric tumors, and a specific discussion of the prognosis of patients with cGIST and rGIST is missing. As the view that colonic and rectal cancer were different is increasingly accepted, there is still no conclusion between cGIST and rGIST. Our date showed that there were statistical differences in tumor size, mitotic index, and NIH risk category between cGIST and rGIST (*P* < 0.05). In terms of tumor size, cGIST was most common with 5 cm–10 cm (33.85%), while rGIST was most common with 2 cm–10 cm (over 81%). In addition, both our findings and the findings presented in Zhu's study (14) showed that the incidence of rGIST with a diameter over 10 cm is significantly lower than that of cGIST with a diameter over 10 cm. The size of GIST is considered to be an important factor affecting postoperative local recurrence and the long-term prognosis of GIST patients (36, 37). However, the existing guidelines all use a uniform critical point (such as 2 cm, 5 cm, or 10 cm) to classify the size of GIST. Whether this classification standard applies to various subtypes of GIST is still unknown. In another study performed in our center (38), the definition criteria of Large-rGIST (L-rGIST), and 5.5 cm were discussed and were deemed an appropriate cut-off value for L-RGIST. Such patients usually showed a male predominance (67.59%), a younger age at onset (56.61 years), a higher operative difficulty, and a poorer prognosis. In recent years, the concept of small GISTs has attracted the attention of most experts in this field (39). A small GIST refers to GIST with a diameter less than 2 cm, and most of these patients have no clinical symptoms. Indeed, many patients are occasionally found in surgery or gastrointestinal endoscopy-it is difficult to distinguish them from other submucosal tumors. Although most small GIST is benign or indolent, a small number of cases have shown aggressive behavior, especially those with a mitotic count >5/5 mm^2^. Currently, there are only a few studies on small GIST from the colon and rectum. Our study showed that the incidence of small GIST in cGIST was slightly higher than that in rGIST (21.5% vs.14.5%). According to the NIH risk category, GIST is divided into two grades based on the mitotic index. GIST with a mitotic ratio lower than 5/50HPF is considered a low grade, while a mitotic ratio higher than 5/50HPF is considered a high grade. The evaluation of the mitotic index of GIST is crucial for risk classification and even prognosis assessment, but in previous studies, not much attention has been paid to it (14). Our study was the first to compare mitotic indices in cGIST and rGIST patients. Compared with rGIST patients, a higher proportion of cGIST cohorts with a high mitotic index and a higher NIH risk rating were detected.

Our study showed that the pathological features of cGIST and rGIST are consistent with those previously reported (12, 13). Similar to most GIST, the majority of stromal tumors in cGIST and rGIST originated from spindle cells. Regarding tumor growth patterns, cGISTs were mainly extraluminal, while rGISTs were mostly intraluminal. cGIST and rGIST patients can present with abdominal pain, mass, bleeding, obstruction, or can be asymptomatic. The clinical manifestations of patients can be affected by a variety of factors, such as tumor size, location, and growth mode. In our study, approximately one-third of cGISTs cases were asymptomatic (11/36), while rGISTs were often causing hemorrhage (32/82), abdominal pain (22/82), and obstruction (21/82).

Since GIST is not sensitive to radiotherapy and chemotherapy, non-operative treatment of GIST mainly relies on targeted therapy with imatinib as the first-line drug. Targeted therapy for GIST is mainly divided into adjuvant therapy and neoadjuvant therapy. The former is mostly used to reduce the postoperative recurrence and metastasis of GIST, and the latter is mostly used to shrink the tumor, reduce intraoperative bleeding, narrow the surgical scope, and convert some unresectable tumors into resectable tumors. Our study showed that the proportion of postoperative adjuvant therapy in cGIST patients was significantly higher than that in rGIST (93.6% vs. 51.6%), which may be related to the higher incidence of lymph node metastasis and postoperative local recurrence in cGIST patients compared with GIST patients at other sites (28). Regarding neoadjuvant therapy, the proportion of patients with rGIST was significantly higher than those with cGIST (49.4% vs. 7.4%). This may be related to differences in the anatomy around rGIST (especially large low rGIST) and cGIST. In another study from our center, mentioned above (38), it was found that rGIST is mostly located in the lower rectum, followed by the middle rectum, and these patients are less likely to undergo primary resection. In addition, preoperative neoadjuvant therapy can be used to shrink rGIST, improve the negative rate of surgical margins, reduce the incidence of surgical complications and the rate of combined organ resection, and improve the anal preservation rate.

Surgery is the only approach to treat GIST. R0 surgery should be considered for localized and resectable cGIST and rGIST (29). Lymph node dissection and mesentery resection are not recommended given the low incidence of lymph node metastasis and skip metastasis in GIST. In most instances, local excision is an effective therapeutic method for GIST (40). However, there is no consensus on whether local resection or radical surgery should be performed for cGIST and rGIST, especially for large low rGIST. Our data showed that local resection was the main surgical method in the two patient groups, and the proportion of rGIST patients undergoing local resection was lower than that of cGIST patients (60% vs. 81%). We speculate that this phenomenon is related to the complex anatomical structure around the rectum and the narrow surgical space of the pelvis, which makes local resection of rGIST difficult and increases the high recurrence rate. Therefore, more aggressive and extensive surgical resection may be helpful to improve the prognosis of rGIST patients (41). We believe that with the introduction of neoadjuvant targeted therapy and transanal endoscopic surgical approaches, an increasing number of rGIST patients will undergo safe and complete local resection.

The location of the tumor is considered an important factor affecting the prognosis of GIST patients. Existing studies have shown that GIST arising from the stomach has been considered a good prognostic feature (42, 43). However, whether there is a difference in prognosis between cGIST and rGIST is still unknown. Our data showed that there was a statistically significant difference in OS between the cGIST group and the rGIST group before PSM (*P* < 0.01). In addition, the OS of rGIST patients was better than that of cGIST patients. Interestingly, no statistically significant differences in OS and PFS were observed between cGIST and rGIST patients after PSM (*P* > 0.05). This phenomenon suggests that prognostic confounding factors will affect the accuracy of the results—tumor entities of cGIST and rGIST might not be different. By including enough indicators related to GIST prognosis and eliminating statistical differences between groups, the results after PSM are similar to the results of a randomized controlled trial. Zhu et al. (14) compared the difference in prognosis between cGIST and rGIST patients. Their results showed that patients with rGIST had a longer OS than patients with cGIST (mean survival 85.7 months vs. 71.3 months, *P* < 0.0001). The study included 398 cGIST and 393 rGIST patients from the National Cancer Database (NCDB) and is the largest study to date. However, due to the large lack of data on prognostic factors of GIST in the NCDB, this study used propensity matching according to the baseline characteristics of patients, which does not exclude the influence of some interfering factors related to prognosis (such as tumor size, mitotic index, NIH risk grade, and growth style). This can be proven by the fact that our results before matching are the same as the results of this study.

The present study has some limitations. Firstly, due to the low incidence of cGIST and rGIST, the sample size of patients included in the study is not large. Therefore, we included patients from literature sources, which may affect the effect of reliability and validity of the results. Secondly, the long-term prognosis data of patients are incomplete, and it is impossible to further compare the metastasis and local recurrence of cGIST and rGIST. In addition, the usage of imatinib in PSM was not included, which may impact the long-term survival of the two groups. Finally, our study is a retrospective analysis, some intraoperative information, postoperative information, and tumor characteristics may not be observed and recorded in detail, such as the gene mutation type. In the future, a randomized controlled trial for comparison will be performed.

## Conclusion

In summary, 65 cGIST and 165 rGIST patients were enrolled in this study, suggesting significant differences in baseline characteristics, clinicopathological features, and OS between cGIST and rGIST patients. However, the difference in anatomical location did not lead to a difference in long-term survival after PSM. Randomized controlled trials with a larger sample size are needed to compare the differences between the two groups.

## Data Availability

The original contributions presented in the study are included in the article/Supplementary Materials, further inquiries can be directed to the corresponding author/s.
