# Supplementary material for: Clinicopathological features and prognosis of colonic and rectal gastrointestinal stromal tumors: A propensity score matching analysis
Source: Front Surg. 2022 Oct 21;9:968585. doi: 10.3389/fsurg.2022.968585 (PMC9634480; doi:10.3389/fsurg.2022.968585)
Supplement: Supplementary file 1 [file Table1.docx]

| **Supplementary Table 1** Guidelines for Reporting Propensity Score Analysis | | |
| --- | --- | --- |
| Section/Topic | Location | Recommendation |
| Title/Abstract | P1L2 | Indicate the use of propensity analysis with a commonly used term in the title or the abstract. |
| Methods |  |  |
| Bias | P5L7 | Describe how propensity score analysis was used to address bias. |
| Statistical analysis | P4L40 | Describe all the analytic methods, including the propensity score methods. |
|  | P5L10 | Indicate the model used to estimate propensity score. |
|  | P5L18 | State the variables included in the propensity score model. |
|  | P5L11 | Explain the variable selection procedure for propensity score model. |
|  | P5L17 | Explicitly state the matching algorithm and distance metric, indicate matching ratio (1:m matching), indicate whether sampling with or without replacement was used, describe the statistical methods for the analysis of matched data, report the package used to create matched sample, and describe methods for assessing the comparability of baseline characteristics in the matched groups. |
|  | P5L20 | Explain how assumption of propensity score analysis was examined. |
|  | P5L19 | Explain how missing data in propensity score estimation were addressed. |
| Results |  |  |
| Participants | P6L31 | Report the sample size for each treatment group before and after matching. |
| Patient characteristics | P6L11 | Describe the distribution of baseline characteristics for each group before propensity score analysis. |
|  | P6L32 | Describe the distribution of baseline characteristics in the matched/weighted groups or in each stratum, and describe the results of the comparability of baseline characteristics. |
|  | P6L31 | Indicate number of patients with missing data for each variable of interest, especially the variables used in propensity score model |
| Main results | P6L31 | Give propensity score analysis estimates and their precision, eg, 95% confidence interval. |
|  | NA | If applicable, give unadjusted estimates and/or adjusted estimates and their precision, eg, 95% confidence interval, and make clear which additional factors were adjusted for. |
| Discussion |  |  |
| Interpretation | P8L33 | Discuss whether imbalance of baseline characteristics still exists, and give a cautious interpretation |
| Generalizability | P8L33 | Discuss the possibility and potential influence of incomplete matching, especially the studies in which the matched sample size is less than 50%. |
